# Highly Pure and Expandable PSA-NCAM-Positive Neural Precursors from Human ESC and iPSC-Derived Neural Rosettes

**DOI:** 10.1371/journal.pone.0039715

**Published:** 2012-07-20

**Authors:** Dae-Sung Kim, Dongjin R. Lee, Han-Soo Kim, Jeong-Eun Yoo, Sung Jun Jung, Bo Young Lim, Jiho Jang, Hoon-Chul Kang, Seungkwon You, Dong-Youn Hwang, Joong Woo Leem, Taick Sang Nam, Sung-Rae Cho, Dong-Wook Kim

**Affiliations:** 1 Department of Physiology, Yonsei University College of Medicine, Seodaemun-gu, Seoul, Korea; 2 Brain Korea 21 Project for Medical Science, Yonsei University College of Medicine, Seoul, Korea; 3 Center for Cell Therapy and Department of Laboratory Medicine, Yonsei University College of Medicine, Seoul, Korea; 4 Department of Physiology, College of Medicine, Hanyang University, Seoul, Korea; 5 Division of Pediatric Neurology, Department of Pediatrics, Severance Children’s Hospital, Yonsei University College of Medicine, Seoul, Korea; 6 College of Life Sciences and Biotechnology, Korea University, Seoul, Korea; 7 CHA Stem Cell Institute, CHA University College of Medicine, Seoul, Korea; 8 Department and Research Institute of Rehabilitation Medicine, Yonsei University College of Medicine, Seoul, Korea; 9 Severance Biomedical Research Institute, Yonsei University College of Medicine, Seoul, Korea; Instituto Butantan, Brazil

## Abstract

Homogeneous culture of neural precursor cells (NPCs) derived from human pluripotent stem cells (hPSCs) would provide a powerful tool for biomedical applications. However, previous efforts to expand mechanically dissected neural rosettes for cultivation of NPCs remain concerns regarding non-neural cell contamination. In addition, several attempts to purify NPCs using cell surface markers have not demonstrated the expansion capability of the sorted cells. In the present study, we show that polysialic acid-neural cell adhesion molecule (PSA-NCAM) is detected in neural rosette cells derived from hPSCs, and employ PSA-NCAM as a marker for purifying expandable primitive NPCs from the neural rosettes. PSA-NCAM-positive NPCs (termed hNPC^PSA-NCAM+^) were isolated from the heterogeneous cell population of mechanically harvested neural rosettes using magnetic-based cell sorting. The hNPC^PSA-NCAM+^ extensively expressed neural markers such as Sox1, Sox2, Nestin, and Musashi-1 (80∼98% of the total cells) and were propagated for multiple passages while retaining their primitive characteristics in our culture condition. Interestingly, PSA-NCAM-negative cells largely exhibited characteristics of neural crest cells. The hNPC^PSA-NCAM+^ showed multipotency and responsiveness to instructive cues towards region-specific neuronal subtypes *in vitro*. When transplanted into the rat striatum, hNPC^PSA-NCAM+^ differentiated into neurons, astrocytes, and oligodendrocytes without particular signs of tumorigenesis. Furthermore, Ki67-positive proliferating cells and non-neural lineage cells were rarely detected in the grafts of hNPC^PSA-NCAM+^ compared to those of neural rosette cells. Our results suggest that PSA-NCAM-mediated cell isolation provides a highly expandable population of pure primitive NPCs from hPSCs that will lend themselves as a promising strategy for drug screening and cell therapy for neurodegenerative disorders.

## Introduction

Proper methods for differentiation and long term culture of neural precursors cells (NPCs) from human pluripotent stem cells (hPSCs) such as human embryonic stem cells (hESCs) and human induced pluripotent stem cells (hiPSCs) are prerequisites for new drug screening and cell-based replacement therapies of the nervous system. In addition, if NPCs have a potential to differentiate into desired subtypes implicated in a disease and are able to be functionally engrafted *in vivo* without outgrowth, these cells and techniques would be more clinically relevant.

Several *in vitro* differentiation protocols are available for generating NPCs from hPSCs [1]–[3]. The recent technical progresses permit us to cultivate an expandable population of NPCs derived from hESCs, which saves time, cost, and labor when maintaining undifferentiated ESCs and differentiating them into NPCs [4]–[9]. Conti *et al.* provided a novel method for propagating NPCs in a monolayer after isolating “neural rosette”-like cells from hESC culture [4]. The neural rosette is the distinctive cellular structure that is morphologically similar to the developing neural tube, arising during the initial neuralization process of ESCs [1], [10]. These cells could be continuously expanded in the presence of basic fibroblast growth factor (FGF) and epidermal growth factor (EGF), and differentiated into neurons and astrocytes *in vitro* as well as *in vivo*. Tabar *et al.* later confirmed that FGF/EGF-expanded NPCs differentiated into oligodendrocytes along with neurons and astrocytes, while migrating extensively according to the migratory plan of developing brain [11]. However, Elkabetz *et al*. claimed that these FGF/EGF-expanded NPCs cannot be regionally specified despite an introduction of patterning cues but progressed toward “definitive” neural stem cells. Instead, they characterized a novel “primitive” neural stem cell stage termed “neural rosette cells” expressing the specific set of early neuroectodermal markers and having an ability to differentiate into various types of region-specified neuronal cells in response to appropriate developmental cues [10]. Although the neural rosette cells have a versatile differentiation profile, they showed tumorigenic behavior after transplantation due to this primitive nature, and no further studies were performed to evaluate their potential for long-term culture. Recently, Koch *et al.* succeeded in partially answering this question by deriving long-term self-renewing neural stem cells with the characteristics of neural rosette cells from hESCs [5]. These cells responded to regional cues inducing region-specific neuronal subtypes, and showed successful functionality of *in vivo* integration without tumor formation.

Despite the enormous progress made in derivation of long-term expandable NPCs, most studies have focused on a strategy of mechanical isolation of the neural rosette [5]–[8]. However, mechanical isolation relies solely on the distinctive morphology of the neural rosette which may be inefficient or generate a heterogeneous population of neural cells that contains undefined derivatives and remnants of undifferentiated hESCs. Furthermore, cultures of early hESC-derived neural rosettes possibly contain neural crest-lineage progenitors [8], [10]. Hence, a strategy to obtain a highly homogeneous population of NPCs should be established to guarantee the highly efficient differentiation rate of desired subtypes and to provide reliable results from drug screening. For these reasons, an isolation method based on the molecular signature is required to ensure homogeneity of primitive NPC cultures. Current attempts to identify specific surface molecules for isolating hESC-derived NPCs achieved considerable outcomes; however, whether the isolated cells are able to propagate stably or to have a diverse differentiation potential is not fully addressed [12].

Polysialic acid-neural cell adhesion molecule (PSA-NCAM) is a homophilic binding glycoprotein preferentially expressed in neural cells having multiple roles in cellular recognition and adhesive processes [13]. Previous studies took an advantage of PSA-NCAM expression to isolate neuronal-restricted precursors from ESC-derived neural derivatives [14]–[15], since this is a well-established marker for immature neuronal cells [16], [17]. In this study, however, we detected PSA-NCAM expression in neural rosette cells during neural differentiation of hESCs, which allowed us to use it as a marker for isolating the primitive NPCs. Using magnetic-based cell sorting technique, we enriched NPCs expressing PSA-NCAM as well as Sox1, Sox2, Nestin and Musashi1, and excluded unwanted cells such as neural crest-lineage progenitors from the culture. In addition, we provide the culture condition for long-term propagation of PSA-NCAM-expressing NPCs (termed hNPC^PSA-NCAM+^) while retaining the cellular and molecular signatures of neural rosette cells and their extensive differentiation potentials. Finally, our *in vivo* analysis showed that hNPC^PSA-NCAM+^ well integrated into the host parenchyma and differentiated into the major three neural lineages without non-neural derivatives and particular signs of tumorigenesis. Our work suggests that PSA-NCAM-targeted cell isolation enables enrichment of a homogenous population of primitive NPCs from hPSCs and minimize safety concerns after transplantation. This provides a promising cell source for practical applications, such as drug screening and regenerative medicine for central nervous system (CNS) diseases.

## Materials and Methods

### Culture and Differentiation of Human PSCs

Human ESCs [H9 (WA09, P35–45), were obtained from WiCell Inc., Madison, WI, USA] [18] and previously established hiPSCs [19] were cultured in hESC medium composed of DMEM/F12 medium (Invitrogen, Carlsbad, CA, USA) supplemented with 20% Knockout-Serum Replacement (Invitrogen), 1× non-essential amino acid (Invitrogen), 0.1 mM beta-mercaptoethanol (Sigma, St. Louis, MO, USA), and 4 ng/ml of basic FGF (Peprotech, Rocky Hill, NJ, USA) as described previously [19]–[20]. To induce neural rosettes, embryoid bodies (EBs) were cultured for 4 days in suspension with 5 µM dorsomorphin (DM) (Calbiochem, San Diego, CA, USA) and 5–10 µM SB431542 (SB) (Sigma) in hESC medium deprived of basic FGF, and then attached on Matrigel-coated dishes (BD Biosciences, Bedford, MA, USA) in 1× N2 (Invitrogen) media supplemented with 20 ng/ml basic FGF for an additional 5 days [21]. Neural rosettes that appeared in the center of attached EB colonies were carefully isolated using pulled glass pipettes from the surrounding flat cells, and then small rosette clumps were seeded on Matrigel-coated dishes after gentle trituration, and cultured in DMEM/F12 supplemented with 1× N2, 1× B27 (Invitrogen) (referred to as N2B27 medium) plus 20 ng/ml basic FGF. Upon reaching approximately 90% confluence, cells were dissociated into single cells by incubation with Accutase (Millipore, Billerica, MA, USA), and PSA-NCAM-positive cells were isolated through magnetic separation system (Miltenyi Biotec, Bergisch Gladbach, Germany) (see below). Isolated PSA-NCAM-positive cells were re-plated on the culture dish at the density of 4 ∼5×10^5^ cells/cm^2^ in N2B27 medium or 1× N2, 0.5× B27 and 0.5× G21 supplement (Gemini Bio-Products, West Sacramento, CA, USA) (referred to as NBG medium) plus 20 ng/ml of basic FGF. The PSA-NCAM-negative cells were also cultured in the same condition as the culture of PSA-NCAM-positive other than the density of 1×10^5^ cells/cm^2^. Culture medium was changed every day and cells were passaged every 2–3 days. If cells did not display sufficient purity, an additional round of cell sorting was performed to enhance the homogeneity of hNPC^PSA-NCAM+^. For further differentiation, hNPC^PSA-NCAM+^ were seeded at a low density (>2×10^4^ cells/cm^2^) and cultured in differentiation media for 3 weeks. Differentiation media was composed of N2B27 or NBG medium supplemented with 0.1% fetal bovine serum (Invitrogen) and 200 µM of ascorbic acid (Sigma). Media changes were performed every two days. For differentiation to midbrain dopaminergic (DA) neurons and spinal motor neurons, hNPC^PSA-NCAM+^ were treated with 200 ng/ml Shh (R&D Systems, Minneapolis, MN, USA) and 100 ng/ml FGF8 (Peprotech) or 100 ng/ml Shh and 0.5 µM retinoic acid (RA) (Sigma) for 7 or 8 days, respectively. The cells were re-plated on new Matrigel-coated dishes at a low density and cultured in differentiation media supplemented with 10 ng/ml brain-derived neurotrophic factor (BDNF) (Peprotech), and 10 ng/ml glial cell-derived neurotrophic factor (GDNF) (Peprotech) for 3 weeks.

### Isolation of PSA-NCAM-positive Cells by MACS

Expanded neural rosette cells (at 80∼90% confluence) were exposed to 10 µM of Y27632 (Sigma) for one hour to prevent cell-death prior to being subjected to MACS procedure. After dissociation using Accutase (Millpore), the cells (∼1×10^8^ cells) were briefly blocked in 1% bovine serum albumin (BSA) (Bovogen, East Keilor, VIC, Australia)-phosphate buffered saline (PBS) solution, and then incubated with anti-PSA-NCAM antibody conjugated with micro-beads (Cat. No. 130-092-966, Miltenyi Biotec) for 15 minutes at 4°C. After extensive washing, the cell suspension was loaded on the separation column (LS column) that was attached to a magnetic stand. Negatively-labeled cells which were passed-through during column-washing were collected in a separate tube, and positively-labeled cells that remained in the column were eluted to another tube with culture media after removing the column from the magnetic stand.

### Immunocytochemical Analysis

Cells were fixed in a 4% para-formaldehyde-PBS solution for 30 minutes. After washing with PBS, cells were permeabilized with 0.1% Triton X-100/PBS (only for intracellular markers), blocked with 2% BSA for 1 hour at room temperature, and primary antibodies were introduced at 4°C over-night. Primary antibodies used in our study were as follows: SSEA4 (1∶500, Santa Cruz Biotechnology), Oct4 (1∶200, Santa Cruz Biotechnology, Santa Cruz, CA, USA), Sox1 (1∶200, Millipore), Pax6 (1∶200, DSHB, Iowa, IA, USA), Nestin (1∶1000, Millipore), Sox2 (1∶200, Millipore), PSA-NCAM (1∶300, Millipore), AP2 (1∶100, DSHB), HNK1 (1∶500, Sigma), Tuj1 (1∶1000, Covance, Berkeley, CA, USA), NeuN (1∶200, Millipore), GFAP (1∶300, Millipore), O4 (1∶200, R&D Systems), TH (1∶300, Pel-freez Biologicals, Rogers, AR, USA; or1∶500, Millipore), Nurr1 (1∶300, Millipore), Pitx3 (1∶400, Millipore), HB9 (1∶200, Millipore), HoxB4 (1∶70, DSHB), Ki67 (1∶300, Vision Biosystems, Newcastle, UK), Zo-1 (1∶100, BD Biosciences), PLZF (1∶50, Calbiochem, San Diego, CA., USA), S100β (1∶1000, BD Biosciences). After primary antibody incubation, the appropriate fluorescence-tagged secondary antibodies (Alexa-Fluor®488 or 594, 1∶500, Molecular Probes, Eugene, OR, USA) were used for visualization. Cells were treated with DAPI (4′, 6′-diamidino-2-phenylindole, Vector, Burlingame, CA, USA) for 5 minutes to visualize the nuclei. Cell images were captured with Olympus IX71 microscope and DP71 digital camera or Olympus FSX100 system, and positively-labeled cells were counted manually or using an image analysis program (MetaMorph v. 7.17, Molecular Devices, USA) in 7∼10 randomly captured images from each of three independent experiments. Values were expressed as means ± standard error of the mean (s.e.m.). Student t-test or analysis of variance (ANOVA) test was used to determine statistical significances.

### FACS Analysis

In order to evaluate the efficiency of MACS, the cells of PSA-NCAM-positive fraction were plated on a culture dish and after 24 hours, the cells were dissociated and incubated in 1% BSA-PBS solution with anti-PSA-NCAM antibody (1∶300, Millipore) as a primary antibody for 15 minutes at 4°C. After extensive washing, the cells were incubated with Alexa-Flour 488 conjugated anti-mouse IgG (1∶500, Molecular Probe) as a secondary antibody for 10 minutes at 4°C. The positive fraction was evaluated using FACSCaliber (BD Biosciences, Sparks, MD, USA) comparing with a blank control. The proportion of SSEA4-positive cells in the hNPC^PSA-NCAM+^ culture was evaluated through the same procedure. All experiments were repeated at least three times, and the values of positive fraction were expressed as average of repeats.

### Semi-Quantitative and Real-time PCR

Total RNAs were isolated using the Easy-Spin® total RNA purification kit (iNtRON Biotechnology, Seoul, Korea) following the manufacturer’s instructions and then 1 µg of total RNA was reverse transcribed with the iScript® cDNA synthesis kit (Bio-Rad, Hercules, CA, USA). Semi-quantitative RT-PCR was performed using EmeraldAmp® GT PCR master mix (Takara Bio Inc, Shiga, Japan) and the reaction was carried out using the GeneAmp® PCR System 2700 (Applied Biosystems, Foster City, CA, USA) under the following conditions: (step 1) 1 minute at 95°C; (step 2) 32–38 cycles of 60 seconds at 95°C, 60 seconds at 56–60°Cs, and 60 second at 72°Cs; (step 3) final extension for 5 minute at 72°C. Real-time PCR for quantitative analysis of gene expression was performed using SYBR Premix Ex Taq™ (Takara Bio Inc.) and CFX96 Real-Time System (Bio-Rad) was used to carry out the reactions under the following conditions: (step 1) 1 minute at 95°C; (step 2) 40–45 cycles of 20 seconds at 95°C, 20 seconds at 60–63°C, and 20 seconds at 72°C; (step 3) final extension for 1 minute at 72°C. Expression values (Ct values) of specific marker genes were normalized according to those of β-actin, and then the levels of normalized expression of the markers were compared between the treated samples and the control samples based on the ΔΔCt method. Relative expression was expressed as means ± s.e.m. obtained from three independent experiments. The primer sequences are provided in Tables S1.

### Electrophysiology

Whole-cell patch-clamp recordings from differentiated neurons from hNPC^PSA-NCAM+^ were performed at room temperature (23±1°C) in normal Tyro de’s solution of the following composition (in mM): 140 NaCl, 5 KCl, 2 CaCl_2_, 1 MgCl_2_, 10 glucose, and 10 HEPES, adjusted to pH 7.4 with NaOH. Differentiated cells were obtained by seeding hNPC^ PSA-NCAM+^ onto Matrigel-coated 12 mm round coverslips and differentiating for 3 weeks. Whole-cell currents of these neurons were recorded using an EPC-10 amplifier (HEKA Electronik, Lamprecht, Germany). Patch pipettes were made from borosilicate glass (Sutter Instrument Company, Novato, CA, USA) using a PC-10 puller (Narishige Co., Tokyo, Japan) and had resistances of 4–6 MΩ when filled with standard intracellular solutions contained (in mM) 130 K-gluconate; 8 NaCl; 0.5 EGTA; 10 HEPES; 4Mg-ATP; 0.3 Na-GTP, adjusted to pH 7.3 with KOH, and 280 mOsm. The solution flow was driven by gravity (flow rate, 10 ml/min). Data were filtered at 3 kHz, digitized at 10 kHz, and analyzed using Pulse program ver.8.67 (HEKA Electronik) and Origin 6.1 software (MicroCal, Northampton, MA, USA).

### Ethics Statement

All experiments were conducted under the supervision of the Department of Laboratory Animal Medicine, Medical Research Center, Yonsei University College of Medicine and following the guidelines of the Institutional Animal Care and Use Committee (Permit No. 2011-0087-1).

### Animals, Cell Transplantation, Tissue Processing, and Analysis

Adult female Sprague-Dawley rats (n = 18) weighed 200–250g at the time of transplantation (Orient Bio Inc., Seongnam, Korea). Neural rosette clumps (unsorted and non-propagated) and hNPC^PSA-NCAM+^ were dissociated using Accutase (Millipore) and suspended to a final cell concentration of 100,000 cells/µL. A total of 2 µl cell suspension was stereotaxically transplanted per rat into a single site at the following coordinates: AP −0.03, ML +0.30, DV −0.55. Four or twelve weeks post-transplantation, rats were deeply anaesthetized with 25% urethane and transcardially perfused with 0.9% saline followed by 4% paraformaldehyde. The brains were post-fixed overnight and cryoprotected in 30% sucrose. Using a cryostat, serial coronal sections were cut at 15∼20 µm and a series of sections were subjected to the standard hematoxyline and eosine (H/E) staining procedure or processed for visualization of targeted markers. Immune-fluorescent staining of brain tissue sections was done as follows: after blocking in a PBS solution containing 0.03% Triton X-100 (Sigma) and 3% bovine serum albumin, tissues were then incubated with the appropriate primary antibodies overnight. The following antibodies were used: human nuclear antigen (HNA) (1∶100, Millipore), Tuj1 (1∶1,000, Covance), NG2 (1∶200, Millipore), GFAP (1∶300, Millipore), TH (1∶300, Pel-freez), GABA (1∶1000, Sigma), and Ki67 (1∶50, Vision Biosystems). Fluorescent secondary antibodies were used for visualization. The proportion of proliferating cells in the grafts was examined in the brain sections stained with anti-Ki67, anti-HNA and DAPI. Five sections that were positive for HNA-immunoreactivity were randomly selected from both hNPC^PSA-NCAM+^ (n = 3) and neural rosette cell (n = 3) transplanted rats. Within each selected section, five images were randomly captured via digital camera attached fluorescence microscope and Ki67-, HNA-, and DAPI-immunoreactive cells within captured images were counted using MetaMorph software program (MetaMorph, Molecular Devices, USA). Values were expressed as means ± s.e.m. of percentages of Ki-67-positive cells and student t-test was used to determine statistical significance (p<0.05) between groups grafted with neural rosette cells and PSA-NCAM-positive cells.

## Results

### Efficient Induction of Neural Rosette Cells from hESCs and Isolation of PSA-NCAM-positive Cells from Neural Rosette Cell Culture using Magnetic-activated Cell Sorting (MACS)

**Figure 1:**
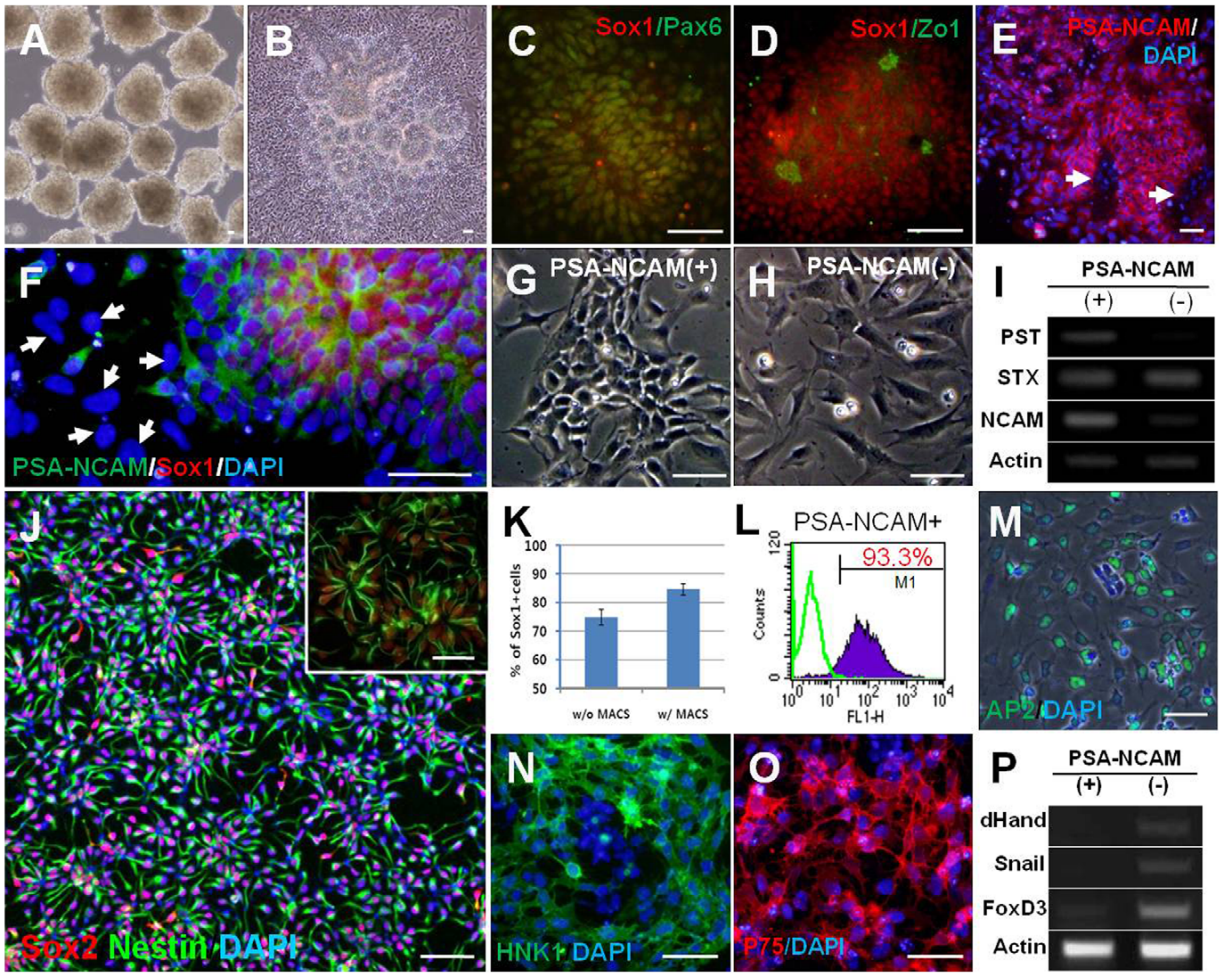
Efficient induction of neural rosette cells and isolation of hNPC^PSA-NCAM+^. (A) hESC derived-EBs treated with dorsomorphin (DM) and SB431542 (SB), (B) When EBs treated with DM and SB were attached onto the Matrigel-coated dish, a large number of rosette structures formed in the center of the colonies after 4–5 days. (C–D) Typical neural markers such as Sox1 and Pax6 were strongly expressed in neural rosettes (C) with strong expression of Zo-1 in the central lumens of rosettes (D). (E–F) PSA-NCAM was highly expressed on the surface of neural rosette cells, but not on the cells out-migrating from the rosette clump (indicated with white arrows in E and F). (G–H) PSA-NCAM-positive cells showed typical morphology of NPCs (G), whereas PSA-NCAM-negative cells had flat and large cell bodies, similar to neural crest cells (H). (I) NCAM and PST, a polysialylating enzyme, were more abundantly expressed in PSA-NCAM-positive fraction after cell sorting. (J) A representative image for the culture of hNPC^PSA-NCAM+^_._ After cell sorting, most of the cells were Sox2/Nestin double-positive, indicating a highly homogeneous culture of NPCs (J, inset) hNPC^PSA-NCAM+^ spontaneously formed the neural rosette structure. (K–L) After sorting, the proportion of Sox1-positive cells and PSA-NCAM-positive cells were enriched up to ∼85% and ∼93% of the total cells, respectively. (M-P) Cells in PSA-NCAM negative-fraction were predominantly positive for AP2 (M), HNK1 (N), and P75 (O) which were used to identify neural crest cells, and enhanced the expression of several genes implicated in neural crest development, such as *dHand*, *Snail*, and *FoxD3* (P). Scale bars: 50 µm.

We previously reported that the simultaneous inhibition of bone morphogenetic protein (BMP) and activin/nodal signals by the small molecules, dorsomorphin (DM) and SB431542 (SB) during embryoid body (EB) culture facilitated neural differentiation in the various hPSC lines [21]. In agreement with previous results, hESC-derived EBs treated with DM and SB 4 days (Fig. 1A) gave rise to a large number of neural rosette structures after attachment culture (Fig. 1B). The number of Pax6-immunoreactive cells significantly increased in the group treated with DM and SB compared to the non-treated control group (Supplementary figure 1). In addition to Pax6, the other neural markers, such as Sox1 and PSA-NCAM were strongly expressed in neural rosettes with pronounced expression of Zo-1, a tight junction protein in the central lumens of rosettes (Fig. 1C–E). To expand neural rosette cells, we mechanically isolated neural rosette clumps under the microscope with caution to avoid flat cells surrounding the neural rosettes, and seeded neural rosette clumps onto the Matrigel-coated culture dish after mechanical triturating. In conditions supplemented with basic FGF, neural rosette islands grew extensively maintaining typical rosette-like morphology and showed marker expressions devoid of detectable contamination by undifferentiated ESCs (Nanog-positive) or other lineage cells (Brachyury- or alpha-fetoprotein-positive cells; Supplementary figure 2). However, we observed heterogeneity to some extent in subsequent propagation of mechanically isolated neural rosette cells. Sox1-negative or P75-positive (presumably neural crest cells) flat and large cells frequently emerged in the periphery of rosette colonies (Supplementary figure 3). In spite of repetitive trials with great caution in isolating neural rosettes, such heterogeneity was repeatedly observed.

**Figure 2:**
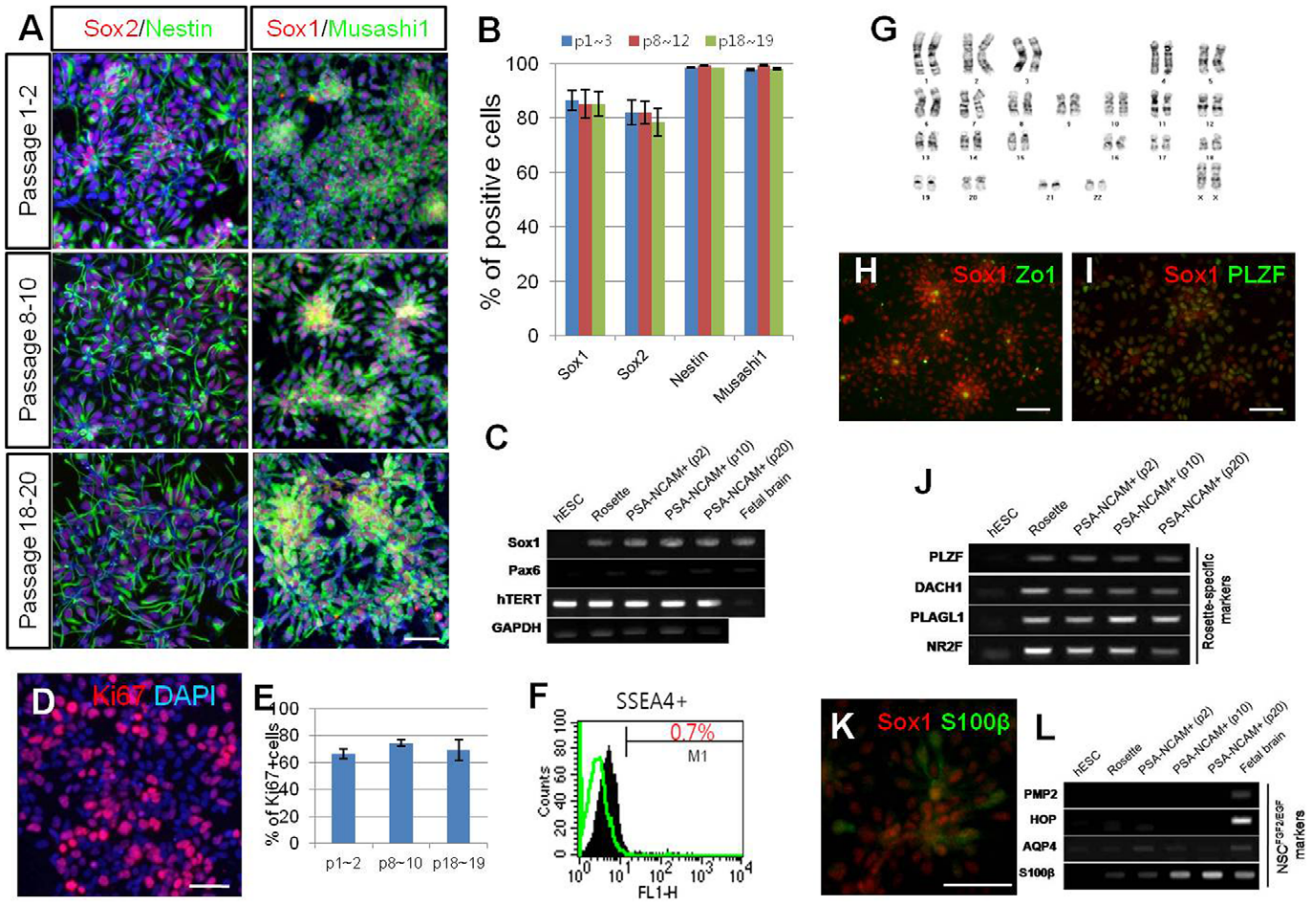
Maintenance and characterization of hNPC^PSA-NCAM+^. (A–B) Neural markers (Sox1, Sox2, Nestin, and Musashi1) were extensively and consistently expressed throughout multiple passages. (C) RT-PCR analysis shows that the expression of *Sox1* and *Pax6* were sustained from the neural rosette stage to hNPC^PSA-NCAM+^; furthermore, *hTERT* was continuously expressed throughout the maintenance culture. (D–E) During multiple passages, about 70% of cultured cells were positive for Ki67, supporting their self-renewing potential. (F) At passage 9, SSEA4-positive cells were rarely detected. (G) hNPC^PSA-NCAM+^ exhibited a normal karyotype at passage 20. (H–J) Along with Zo-1 expression in the lumen-side of the rosette structure (H), co-expression of PLZF with Sox1 (I) as well as prominent expression of “neural rosette-specific” genes (J) indicate that hNPC^PSA-NCAM+^ exhibited the characteristics of typical neural rosette cells. (K–L) Although NPs did not express either *PMP2* or *HOP*, they did express *AQP4* and *S100β* to some extent, which were proposed to be specifically expressed in FGF/EGF-expanded neural cells (NPC^FGF/EGF^). Scale bars: 50 µm.

**Figure 3:**
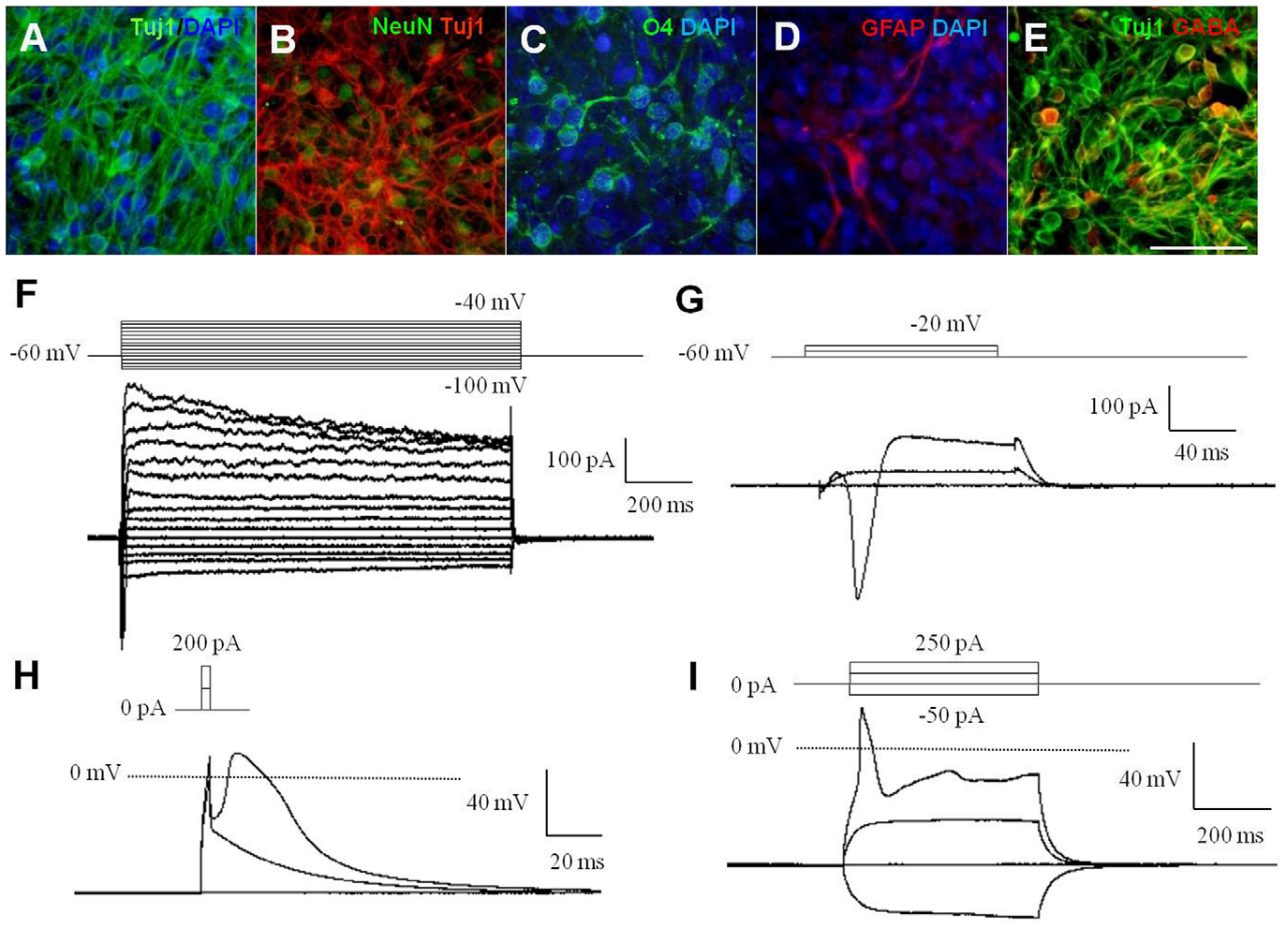
hNPC^PSA-NCAM+^ exhibited multipotency and differentiated neurons were electrophysiologically functional. (A–D) hNPC^PSA-NCAM+^ were spontaneously differentiated into neurons (A­B), oligodendrocytes (C), and astrocytes (D) in the absence of mitogens. (E) GABA-immunoreactivity was frequently observed among Tuj1-positive cells. (F–G) Voltage-dependent membrane currents: depolarizing voltage steps elicited outward K^+^ currents (F) and fast inward Na^+^ currents (G). (H–I) In current-clamp recordings, short (3 ms) or prolonged (500 ms) depolarizing current injections above supra-threshold elicited the single action potential (H), and the fast action potentials (I). Scale bars: 50 µm.

While characterizing the expanded neural rosette cells through immunocytochemical analysis, we made an interesting observation that PSA-NCAM immunoreactivity was extensively detected in Sox1-positive cells of the core of neural rosettes. On the other hand, the immunoreactivity of PSA-NCAM was faint or absent in the cells migrating out of rosette islands (Fig. 1E–F). From this observation, we assumed that PSA-NCAM mediated-cell sorting might enable a pure population of cells consisting of primitive NPCs without Sox1-negative or P75-positive neural crest cells. Thus, we isolated PSA-NCAM positive cells from the expansion culture of neural rosette clumps using anti-PSA-NCAM micro-beads and magnetic-based separation (see Materials and Methods). After cell sorting, PSA-NCAM-positive NPCs (termed hNPC^PSA-NCAM+^ afterward) were highly homogeneous and had a spindle shape, the typical morphology of NPC. More importantly, these cells reassembled into the rosette-like structure at high density [Fig. 1G and J (inset)]. In contrast, negatively sorted cells had flat and larger cell bodies similar to neural crest cells and did not form any intercellular structure (Fig. 1H). Semi-quantitative reverse-transcription polymerase chain reaction (RT-PCR) analysis confirmed that NCAM and Golgi-associated polysialyltransferase, PST, (i.e., ST8SiaIV/PST), the enzyme involved in the polysialylation of NCAM, were more abundantly expressed in the PSA-NCAM-positive fraction than in PSA-NCAM-negative fraction, even if the expression of STX (i.e., ST8SiaIV/STX), the other polysialylating enzyme, did not show a significant difference between the two groups (Fig. 1I). Sox1-positive and PSA-NCAM-positive cells were enriched up to about 85% and 93.3% of the total cells after MACS, respectively (Fig. 1K and L). Careful analysis of negatively sorted cells for PSA-NCAM selection revealed that these cells were predominantly labeled with antibodies against several neural crest markers such as AP2, HNK1 and p75 (Fig. 1M–O). RT-PCR analysis also supported that the PSA-NCAM-negative fraction was enriched by cells expressing genes involved in neural crest development such as *dHand, Snail,* and *FoxD3* (Fig. 1P). Collectively, these data suggest that PSA-NCAM targeted cell sorting not only enables the selection of a purified NPC population, but also largely excludes neural crest cells from the expansion culture of neural rosette cells.

### hNPC^PSA-NCAM+^ were Expanded through Long-term Culture while Retaining the Characteristics of Primitive NPCs

At high density (>5×10^5^ cells/cm^2^), a homogenous population of hNPC^PSA-NCAM+^ were maintained stably as a monolayer without any changes in morphology or marker expression, while continuously passaged every 2 days by enzymatic dissociation. At low plating-density, Tuj1-positive neuronal progenitors were frequently observed (∼20% of total cells at 5×10^4^ cells/cm^2^); however, the proportion of these cells diminished as plating density increased [<4% of the total cells were Tuj1-positive at 5×10^5^ cells/cm^2^ (Supplementary figure 4)], which is consistent with a previous report [10]. Most of the hNPC^PSA-NCAM+^ consistently retained a high level of neural cell marker expression (Sox1, Sox2, Nestin, and Musashi1) and rosette-forming nature through multiple passages (at least 20 passages) (Fig. 2A, B). These cells could be cryopreserved in liquid nitrogen for several months, and cultured well after thawing without losing neural characteristics (data not shown). Immunocytochemical analysis with an anti-Ki67 antibody reveals that about 70% of the total cells were proliferative in the presence of basic FGF independent of passage number (Fig. 2D and E). This is consistent with the sustained expression of *hTERT*, a telomere lengthening enzyme (Fig. 2C). Furthermore, these cells exhibited a normal karyotype at passage 20 (Fig. 2G). SSEA4-positive cells, which are regarded as the potential undifferentiated cells, were 0.7% of the total analyzed hNPC^PSA-NCAM+^ at passage nine (Fig. 2F); however, SSEA4-immunoreactive cells detected in hNPC^PSA-NCAM+^ culture did not co-express Nanog (data not shown). Instead, SSEA4-positive cells in hNPC^PSA-NCAM+^ culture often co-labeled with Musashi1 (Supplementary figure 5), implying that they were not the undifferentiated remnants, but might be the early neuroepithelial cells observed in the developing human embryonic CNS [22].

**Figure 4:**
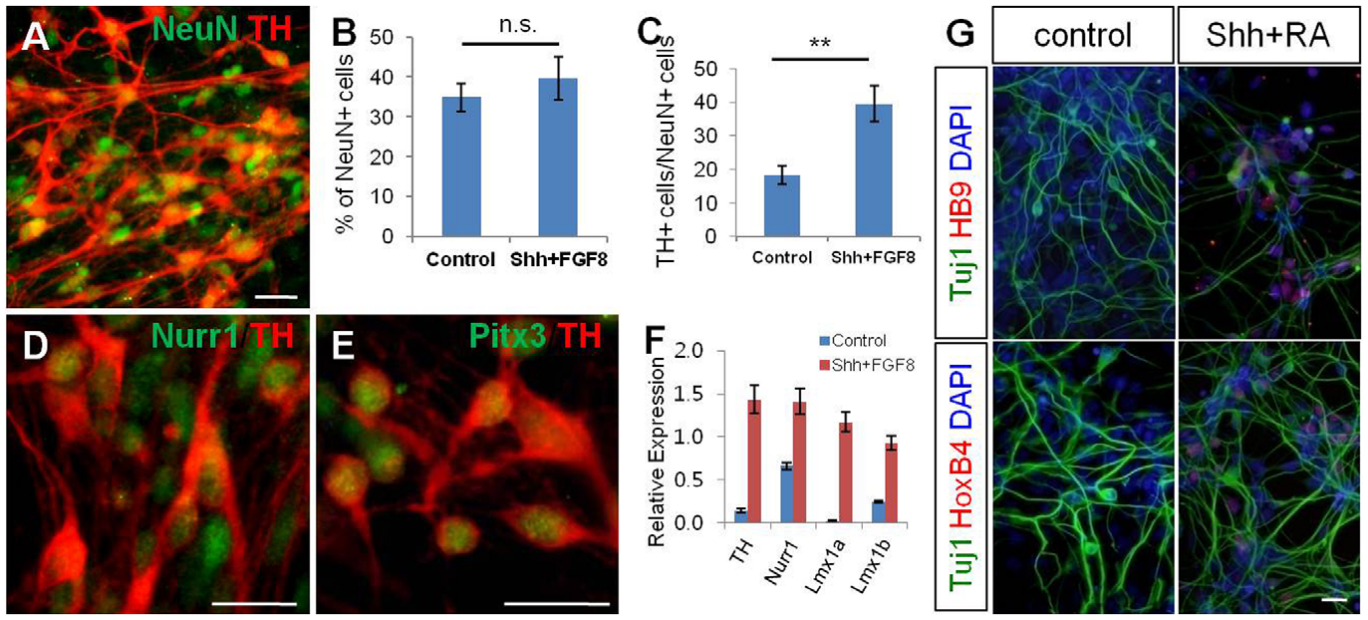
hNPC^PSA-NCAM+^ were able to differentiate into specific neuronal subtypes using regionalizing cues. (A) Neurons exhibiting dopaminergic phenotypes were derived from hNPC^PSA-NCAM+^. (B–C) The cells treated with Shh and FGF8 for 8 days, followed by treatment with BDNF, GDNF, and ascorbic acid gave rise to a larger number of TH-positive neurons than control cells (non-treated group) without a significant change in total neuronal differentiation. (D–E) TH-positive cells co-expressed with Nurr1 and Pitx3. (F) Quantitative RT-PCR analysis confirmed the enhanced expression of several transcription factors involved in dopaminergic differentiation after treatment with Shh and FGF8 to hNPC^PSA-NCAM+^. (G) RA/Shh-exposed hNPC^PSA-NCAM+^ showed induction of HoxB4 and HB9, a posterior gene and a motor neuron-specific gene, respectively. Scale bars: 20 µm, n.s., not significant, **, p<0.01.

**Figure 5:**
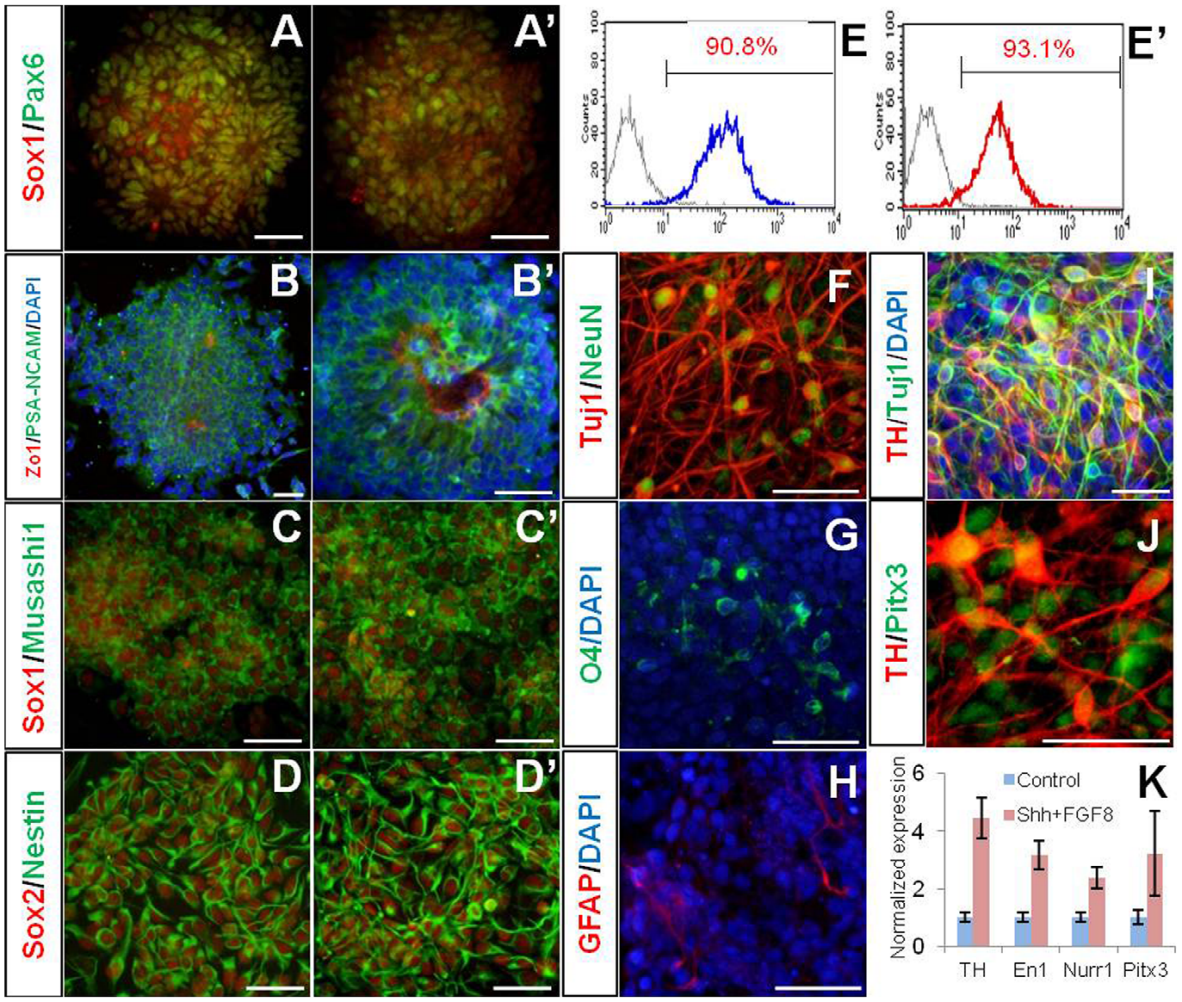
Isolation and differentiation of hNPC^PSA-NCAM+^ from iPSCs; spontaneous and directed differentiation from them. (A–E) Data from WT-iPSC3 and (A’–E’) from PD-iPSC4. (A, A’ and B, B’) Induction of neural rosettes from iPSCs. Neural markers (Sox1, Pax6, and PSA-NCAM) were prominently expressed in rosette structure with distinct Zo-1 expression in lumen. (C–E and C’–E’) hNPC^PSA-NCAM+^ were successfully isolated from two hiPSC lines and maintained in adherent culture with the comparative efficiency to that of hESCs. (F–H) hNPC^PSA-NCAM+^ derived from PD-iPSC were able to differentiate into neurons, oligodendrocytes and astrocytes. (I–K) PD-specific hNPC^PSA-NCAM+^ primed with Shh and FGF8 efficiently gave rise to neurons exhibiting DA neuron phenotypes. Scale bars: 50 µm.

Zo-1 expression in the apical side of cellular architecture and co-expression of PLZF, described as a “rosette-specific marker” [10] with Sox1 indicated that hNPC^PSA-NCAM+^ showed the characteristics of typical neural rosette cells (Fig. 2H and I). In particular, the expression of PLZF was continuously maintained over multiple passages (Fig. 2J and Supplementary figure 6). In addition, other rosette-specific genes, such as *DACH1*, *PLAGL1*, and *NR2F* were strongly expressed through multiple passages as well (Fig. 2J). These data support that hNPC^PSA-NCAM+^ maintain the partial characteristics of neural rosette cells throughout expansion.

**Figure 6:**
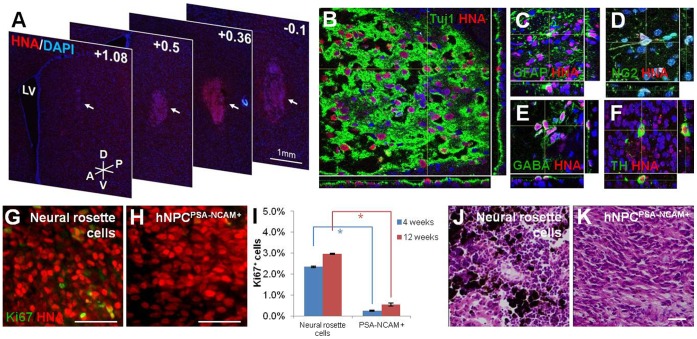
Integration and survival of hNPC^PSA-NCAM+^ in adult rat striatum. (A) A series of coronal sections from +1.08 to −0.1 shows survival and integration of hNPC^PSA-NCAM+^ grafts cells in the host striatum 4 weeks after transplantation. (B) Dual-label confocal immunofluorescence microscopy with DAPI confirms a large population of HNA- and Tuj1-positive cells within the graft of hNPC^PSA-NCAM+^. (C–D) In addition to Tuj1-positive cells, GFAP- and NG2-positive cells within HNA-positive graft sites show the potential of hNPC^PSA-NCAM+^ in differentiating into all three neural lineages *in vivo*. (E–F) Further dual immunohistochemistry for HNA:GABA and HNA:TH indicates the ability of grafted cells to differentiate into various neuronal subtypes. (G–H) A representative area of a neural rosette cell graft indicates a significantly higher number of Ki67-positive cells and clusters whereas only a few Ki67-positive cells with no clusters are observed for hNPC^PSA-NCAM+^. (I) Bars indicate the proportions of Ki67-positive cells in the grafts of neural rosette cells and hNPC^PSA-NCAM+^ at 4 weeks (blue bars) and 12 weeks (red bars) post-transplantation as mean ± s.e.m. (J–K) Representative images of H/E-stained brain tissues grafted with unsorted neural rosette cells (J) and hNPC^PSA-NCAM+^ (K). Melanocyte-like cells stained in dark-brown color were shown only in the grafts of unsorted neural rosette cells (J). Scale bar: 50 µm. Abbreviations: LV, lateral ventricle; A, anterior; P, posterior; D, dorsal; V, ventral. *, p<0.05.

A previous report suggested that FGF/EGF-expanded NPCs, referred to as NSC^FGF/EGF^, were another type of NPCs distinct from the neural rosette-type NPCs in specific marker expression, differentiation potential, and *in vivo* behavior [10]. To address whether hNPC^PSA-NCAM+^ shared gene expression with NSC^FGF/EGF^, we examined NSC^FGF/EGF^-specific gene expression, *PMP2, HOP, AQP4*, and *S100β* in cultured hNPC^PSA-NCAM+^ using RT-PCR. Although we did not observe expression of *PMP* and *HOP* in every step of differentiation, *AQP4* and *S100β* were modestly detected in hNPC^PSA-NCAM+^ (Fig. 2L). Moreover, S100β-immunoreactivity was detected in the cells at passage 10 (Fig. 2K). Altogether, these findings suggest that hNPC^PSA-NCAM+^ share the cellular and molecular characteristics of both neural rosette cells and NSC^FGF/EGF^, implying that hNPC^PSA-NCAM+^ may be the cell in the developmental stage between neural rosette cell and NSC^FGF/EGF^.

### hNPC^PSA-NCAM+^ are Multipotent and Differentiated into Specific Neuronal Subtypes Responsive to Regional Cues

To address whether hNPC^PSA-NCAM+^ could generate the three types of neural lineage cells, we differentiated them in differentiation media (N2B27 or NBG media). Ascorbic acid (200 µM) and fetal bovine serum (0.1% v/v) were supplemented in culture media for better viability of differentiating cells. After 3 weeks, hNPC^PSA-NCAM+^ differentiated into Tuj1 and/or NeuN-positive neuronal cells, O4-positive oligodendrocyte precursors and glial fibrillary acidic protein (GFAP)-positive astrocytes (Fig. 3A–D). Tuj1-positive cells constituted most of the differentiated cells (>90% of total cells), among them, cells showing GABA-immunoreactivity was prevalent (Fig. 3E). O4-positive cells were observed in patches (3–5% of total cells), while GFAP-positive cells were occasionally detected (Fig. 3C and D). These data suggest that hNPC^PSA-NCAM+^ are highly neurogenic.

To explore whether the neurons differentiated from hNPC^PSA-NCAM+^ were biologically functional, their electrophysiological properties were examined. The membrane characteristics of these neurons were determined in the voltage- and current-clamp configuration. The neurons exhibited voltage-dependent membrane currents, which were elicited by 1000-ms or 100-ms voltage steps from −100 to 40 mV from a holding potential of −60 mV in differentiated neurons (Fig. 3F and G). Depolarizing voltage steps elicited both large outward potassium currents (Fig. 3F) and fast inward sodium currents (Fig. 3G). In current-clamp mode, the single action potential of the differentiated neurons was also evoked by short depolarizing current injections above supra-threshold (3 ms), and these neurons fired fast action potentials by prolonged depolarizing current injections (500 ms) (Fig. 3H and I). Consistent with the results in sodium current, the action potential was slowly activated. These results indicate that the differentiated cells have the electrophysiological properties of functional neurons.

Next, we examined whether hNPC^PSA-NCAM+^ could be induced into specific neuronal subtypes by introduction of regional cues. To direct hNPC^PSA-NCAM+^ into the neurons exhibiting midbrain DA phenotypes, we added Sonic Hedgehog (Shh) and FGF8 into the culture for 7–8 days, then induced them into mature neuronal cells with treatment of ascorbic acid, BDNF, and GDNF after withdrawal of mitogens. As a result, we observed that the treatment of Shh and FGF8 significantly increased the number of tyrosine hydroxylase (TH)-positive cells among NeuN-positive neurons compared to the control group, whereas a significant increase of NeuN-positive cells was not observed (Fig. 4A–C). Many of the TH-positive neurons generated by treatment with Shh and FGF8 expressed Nurr1 and Pitx3, known as the critical transcription factors in DA neuron development (Fig. 4D and E). Furthermore, hNPC^PSA-NCAM+^ strongly expressed the transcripts implicated in the commitment of DA neurons such as Nurr1, *Lmx1a, and Lmx1b* after exposure to Shh and FGF8 (Fig. 4F). To explore whether hNPC^PSA-NCAM+^ could be regionalized into caudal and ventral fates, they were cultured in the absence or presence of RA and Shh for 8 days. Indeed, we observed that hNPC^PSA-NCAM+^ primed by RA and Shh strongly expressed HoxB4, a posterior hindbrain marker, and HB9, a motor neuron marker, compared to control group (Fig. 4G). These data collectively show that the hNPC^PSA-NCAM+^ are multipotent neural stem cells and primitive NPCs preserving the responsiveness to extrinsic regionalizing factors that can generate region-specific neuronal subtypes.

### Isolation and Differentiation of hNPC^PSA-NCAM+^ from hiPSCs

We next addressed whether our protocol was reliable for generating a pure population of hiPSC-derived neural progeny. Previously, we established two hiPSC lines (WT-iPSC3 and PD-iPSC4) by transduction of retroviral vectors carrying Oct4, Sox2, c-Myc, and Klf4 into the normal human dermal fibroblasts and Parkinson’s disease patient-derived fibroblasts and extensively characterized [19]. Upon neural induction with treatment of DM and SB, robust neural rosette structures which were extensively labeled with Pax6, Sox1, PSA-NCAM and Zo-1 antibodies were generated from both cell lines (Fig. 5A and B, A and B́). After subsequent propagation of isolated rosette clumps for 4–5 days, PSA-NCAM-positive cells were isolated by MACS, and then maintained stably as a monolayer culture (Fig. 5C and D, C and D́) showing the comparative efficiency to that of hESCs (Fig. 5E and E ´). In particular, hNPC^PSA-NCAM+^ derived from PD-iPSC4 spontaneously differentiated into Tuj1/NeuN-double positive neuronal cells, O4-positive oligodendrocyte precursors, and GFAP-positive astrocytes under differentiation conditions (Fig. 5F–H). Furthermore, they could be readily biased toward TH-positive neurons exhibiting midbrain dopaminergic phenotypes through the same strategy as hESC differentiation (Fig. 5I–K). Taken together, these data show that our method for induction, isolation, and maintenance of hNPC^PSA-NCAM+^ is applicable to both hESCs and hiPSCs.

### hNPC^PSA-NCAM+^ were Well Integrated and Differentiated in the Rat Brain after Transplantation Showing Limited Proliferation Capacity

To explore the ability of hNPC^PSA-NCAM+^ to differentiate into three major types of neural lineages *in vivo* and to address possible neural outgrowth, 2×10^5^ hNPC^PSA-NCAM+^ at passage 7–10 were stereotaxically transplanted into the adult rat striatum (n = 5), with an equal number of unsorted, non-propagated neural rosette cells as a control group (n = 5). Rats were sacrificed 4 weeks after post-transplantation and the transplanted cells were visualized via human nuclear antigen (HNA) staining (Fig. 6A–H). The graft of hNPC^PSA-NCAM+^ could be observed as a rugby-ball shape with approximately 2 mm on the y-axis and 1 mm on the x-axis and mildly dispersed from the injection site in the host striatum, indicating that the graft survived and integrated into the host striatum (Fig. 6A). HNA-positive cells in the graft site of hNPC^PSA-NCAM+^ were primarily Tuj1-positive; however, occasional GFAP-and NG2- positive cells were located suggesting that hNPC^PSA-NCAM+^ cells were capable of differentiating into all three neural lineages: neurons, astrocytes, and oligodendrocytes *in vivo* (Fig. 6B–D). Furthermore, neuronal subtypes such as GABA- and TH- positive cells were observed within HNA-positive grafts through an additional histological analysis (Fig. 6E–F). To determine whether neural outgrowth would result from continuous proliferation of grafted cells, the number of Ki67-positive cells in hNPC^PSA-NCAM+^ grafted tissues (n = 3) were compared with those of unsorted neural rosette cell grafted tissues (control group, n = 3) (Fig. 6G–H). As shown in Figure 6I, the percentage of Ki67-positive cells in the graft of unsorted neural rosette cells was 2.35±0.02%, while the significantly lower percentage of 0.27±0.04% was measured in hNPC^PSA-NCAM+^ grafts suggesting that 8.7 times fewer proliferating cells were present within hNPC^PSA-NCAM+^ grafts 4 weeks post-transplantation. Additionally, unlike unsorted neural rosette cells, hNPC^PSA-NCAM+^ had no visible clusters of Ki67-positive cells. To further investigate the continuous proliferation of a graft and the possible neural outgrowth, we additionally examined rat brains transplanted with 2×10^5^ hNPC^PSA-NCAM+^ (n = 4) and unsorted neural rosette cells (n = 4) 12 weeks post-transplantation. In spite of the extended period of grafting study, no tumor-like structures and teratoma formations were observed in both groups. However, the occurrence rate of Ki67-positive cells in grafts with unsorted neural rosette cells was still significantly higher than the grafts with hNPC^PSA-NCAM+^ (2.97% ±0.02% vs. 0.54±0.09%) (Fig. 6I), and the clusters of Ki67-positive cells were obvious in the grafts of unsorted neural rosette cells (Supplementary figure 7). Interestingly, melanocyte-like pigmented cells were observed in 2 out of 4 grafts of unsorted neural rosette cells (Fig. 6J), while no such cell was observed in hNPC^PSA-NCAM+^ grafts (Fig. 6K). Melanocytes were known to be originated from neural crest cells [23], the melanocyte-like cells in the graft were presumably differentiated from PSA-NCAM-negative cells contaminated in unsorted neural rosette cells.

Taken together, these data indicate that hNPC^PSA-NCAM+^ cells integrate into the adult mammalian brain, have full capacity of neural differentiation potential *in vivo,* and have a significantly lower possibility of neural outgrowth when compared to unsorted and non-propagated neural rosette cells. Furthermore, it is improbable to generate neural crest-originated non-neural lineage cells in transplantations of hNPC^PSA-NCAM+^ cells.

## Discussion

We previously accomplished the efficient neural conversion from a variety of hPSC lines through treatment with small molecules inhibiting BMP and activin/nodal signals [21]. More importantly, we showed that our approach enabled us to circumvent various potentials of neural differentiation among various hPSC lines. Since the recent report claimed that each hESC line has its own differentiation propensity toward a specific lineage [24] and another report argued that hiPSCs showed a lower and variable efficiency for neural differentiation compared to hESC lines [25], our approach in the initial step of differentiation is beneficial to enhance the efficiency of neural differentiation and the homogeneity of prospective NPC cultivation from hESCs as well as hiPSCs.

A homogeneous population of NPCs can be acquired by a selection procedure. NPCs or more specified progenitors have been isolated from hESCs or human fetal neural tissues by sorting the cells expressing fluorescent proteins under a promoter of specific marker protein [26]–[28], or by immunological methods with antibodies recognizing specific surface antigens [29]–[30]. Although a genetic lineage selection using a marker-restricted expression of fluorescence protein is a widely used method and reliable for lineage-specific isolation, it requires tedious genetic manipulation and such engineered cells seem yet unsuitable for clinical purposes. By contrast, immune-isolation strategy is a relatively simple and comprehensive method for isolating the desired cells from a mixed population and as a result, such a strategy is currently employed for medical applications [31]. Therefore, with an appropriate surface antigen, the immune-isolation approach allows to acquire safe and reproducible hPSC-derived progenies for the future clinical translation.

PSA-NCAM has been considered as a marker representing immature neuronal cells and employed for isolating neuronal-restricted precursors [14]–[15], [32] due to its specific temporal expression and differentiation potentials [33]–[34]. In the present study, however, we showed that PSA-NCAM is expressed in most of hPSC-derived neural rosette cells. Consistently, a previous report demonstrated that rosette clumps derived from hESCs largely contained PSA-NCAM-positive cells [1]. From these data, we inferred that PSA-NCAM-targeted immuno-isolation in a mechanically isolated neural rosette culture could eliminate the remnants of possible non-neural and non-neural rosette cells, hence enhancing the homogeneity of culture. Consequently, PSA-NCAM-targeted sorting enriched Sox1-positive NPCs (Fig, 1K) and elicited highly homogeneous and expandable population of NPCs while retaining a typical morphology as well as partial molecular characteristics of neural rosette cells (Fig. 2). Furthermore, we unexpectedly came across that PSA-NCAM targeted sorting was able to eliminate neural crest-lineage progenitors (Fig. 1M–P). According to a previous report, the cells (or cell clusters) expressing early neural crest markers predominantly located at the periphery of neural rosette islands [35]. In addition, another recent report demonstrated that the cells migrating around the adherent neural rosette clusters exhibited the characteristics of neural crest cells [36]. According to our data, the cells located at the same periphery region of neural rosette islands showed weak or negative PSA-NCAM immunereactivity, and these cells predominantly exhibited the characteristics of neural crest cells. Therefore, we suggest that PSA-NCAM can be not only a marker for purifying the CNS-type NPCs, but it may also be used for a potential marker for discriminating neural crest cells from the early neural derivatives of hPSCs.

It would be advantageous to exclude neural crest cells from NPC culture for various reasons. Since neural crest cells could give rise to non-neural cells such as melanocytes, smooth muscle cells, and chondrocytes [23], [35]–[36], it may be able to hamper efficient differentiation to specific subtypes of neuronal/glial cells. More importantly, if neural crest cells present in neural precursor population give rise to non-neural tissues in CNS after transplantation, it would evoke an unexpected safety problem. Our transplantation data showing the occurrence of melanocyte-like cells in the grafts of unsorted neural rosette cells (Fig. 6J) strongly supported our hypothesis; therefore, PSA-NCAM-mediated cell sorting can be an advantageous strategy to minimize these potential problems by eliminating neural crest cells from a neural precursor population.

Our results also provided the culture conditions for stable propagation of hNPC^PSA-NCAM+^ as a homogeneous monolayer. Distinct from previous reports showing similar results [4]–[9], our culture condition of hNPC^PSA-NCAM+^ did not contain EGF which others required to stably maintain NPCs. Even in the absence of EGF, hNPCs^PSA-NCAM+^ were able to propagate while preserving the initial characteristics of primitive neural precursors throughout several passages. These data suggest that EGF is not likely a critical factor for self-renewal and maintenance of the primitive features of NPCs derived from hPSCs.

Lastly, we assessed the *in vivo* behavior of hNPC^PSA-NCAM+^ in the adult rat striatum. At 4 weeks post-transplantation, hNPC^PSA-NCAM+^ were successfully integrated into the host parenchyma and differentiated into the various types of neurons, oligodendrocytes, and astrocytes without particular signs of neural outgrowth. When compared to the unsorted, non-propagated neural rosette cells, a significantly lower number of proliferating cells was found in hNPC^PSA-NCAM+^ in graft sites. Furthermore, we attained the similar results from a longer period of grafting study (∼12 weeks) as well. Such an observation may be the result of hNPC^PSA-NCAM+^ expressing the partial characteristics of definitive NSC^FGF/EGF^ which may have allowed the grafted cells to avoid neural outgrowth *in vivo*.

In conclusion, we identified that the expression of PSA-NCAM in the neural rosette cells derived from hESCs and cell sorting mediated with PSA-NCAM generated a highly homogeneous population of primitive NPCs while retaining the characteristics of neural rosette cells. During the long-term culture, hNPC^PSA-NCAM+^ maintained the properties of self-renewal and comprehensive differentiation potential like primitive neural stem cells. Since our strategy is also applicable to hiPSCs, it provides an efficient platform for new drug discovery and toxicity testing, a disease model for incurable neurodegenerative disorders, and a reliable cell source for the future cell replacement therapy.

## Supporting Information

Figure S1 **Simultaneous inhibition of BMP and Activin/Nodal signals with small molecules (dorsomorphin and SB431542) during EB culture facilitated neural differentiation of hESC.** H9-derived hEBs were cultured in the absence (A) or presence (B) of dorsomorphin (DM, 5 µM) and SB431542 (SB, 5∼10 µM) for 4 days, and then attached onto the Matrigel-coated culture dish. After 4 days of adherent culture, the cells were dissociated and analyzed by immunocytochemistry with anti-Pax6 antibody. Quantitative analysis showed that the treatment of DM and SB during EB culture significantly increased the number of Pax6-positive NPs among differentiated cells (C). Scale bar: 50 µm.
(TIF)

Figure S2 **Mechanically isolated neural rosette cells were extensively proliferated in the presence of basic FGF.** When hESC-derived neural rosette cells were mechanically isolated and cultured in the presence of basic FGF on culture dishes, the cells vigorously proliferated retaining the expression of neural marker, Sox1 and the typical rosette structure (A–C) devoid of endodermal and mesodermal derivatives (D–E). Scale bars: 100 µm.
(TIF)

Figure S3 **Culture of mechanically isolated neural rosette cells showed the heterogeneity.** When mechanically isolated neural rosette cells were expanded on culture dishes, Sox1-negative cells (presumable non-neural cells, indicated by asterisks) and neural crest-lineage precursors (P75-positive cells, indicated by arrows) occasionally appeared in the culture. Scale bars: 100 µm.
(TIF)

Figure S4 **Relationship between the plating density of hNPC^PSA-NCAM+^ and the occurrence of immature neurons. Relationship between the plating density of hNPC^PSA-NCAM+^ and the occurrence of immature neurons.** (A–B) Representative pictures of immunocytochemical analysis with anti-PSA-NCAM and Tuj1 antibodies at cell density of 1×10^5^ cells/Cm^2^ (A) and 5×10^5^ cells/Cm^2^ (B). At low plating-density, Tuj1-positive neuronal progenitors were frequently observed; however, the portion of Tuj1-positive cells significantly diminished as plating density was increasing (C). Scale bar: 50 µm.
(TIF)

Figure S5 **SSEA4-immunoreactive cells in a hNPC^PSA-NCAM+^ population were in fact the early neuroepithelial cells rather than undifferentiated cells.** Immunocytochemical analysis revealed that SSEA4-positive cells in hNPC^PSA-NCAM+^ culture coexpressed Musashi1 (indicated by arrow) and were under the process of cell division.
(TIF)

Figure S6 **The co-expression of PLZF with Sox1 was maintained from the neural rosette-stage throughout the propagation of hNPC^PSA-NCAM+^.** Scale bars: 50 µm.
(TIF)

Figure S7 **Representative images of hNPC^PSA-NCAM+^ and neural rosette cell grafts 12 weeks post-transplantation expressing immunoreactivity of HNA and Ki67.** While hNPC^PSA-NCAM+^ graft showed minimal and dispersed Ki67-positive expressions, neural rosette cell graft maintained pronounced expressions of Ki67 and the positive cells showed an apico-basal growth pattern (indicated by arrows). Scale bars: 100 µm.
(TIF)

Tables S1 **Primer sets for semi-quantative PCR (Table S1) and primer sets for real-time PCR (Table S2).**
(DOCX)
