# Supplementary figures and images for: Highly Pure and Expandable PSA-NCAM-Positive Neural Precursors from Human ESC and iPSC-Derived Neural Rosettes
Source: PLoS One. 2012 Jul 20;7(7):e39715. doi: 10.1371/journal.pone.0039715 (PMC3401209; doi:10.1371/journal.pone.0039715)

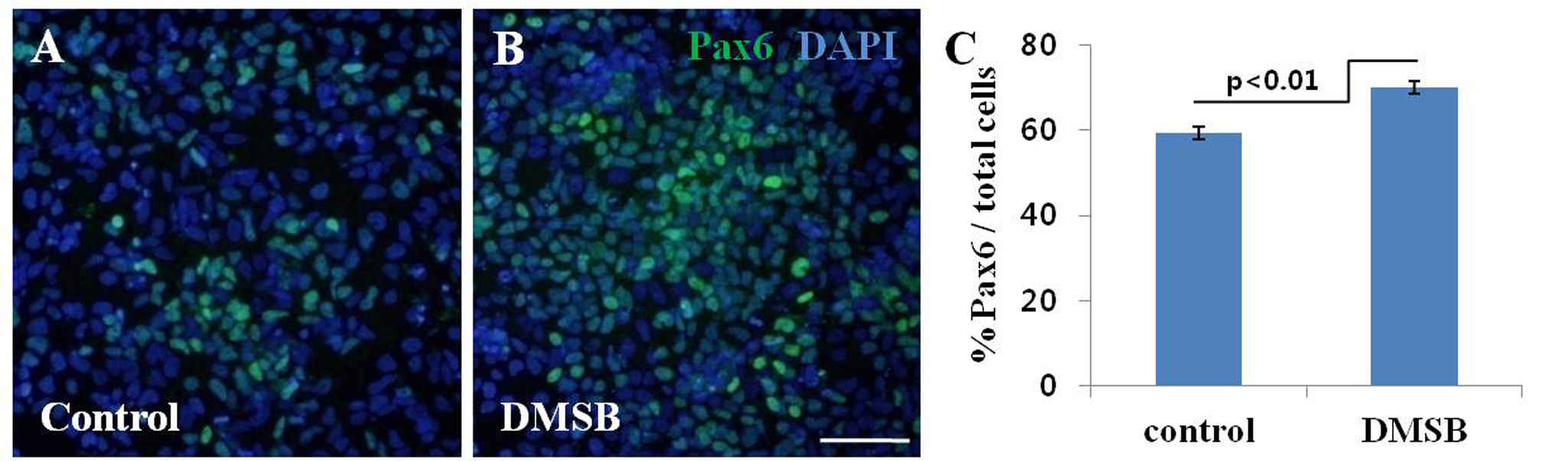

Supplement: Figure S1 — Simultaneous inhibition of BMP and Activin/Nodal signals with small molecules (dorsomorphin and SB431542) during EB culture facilitated neural differentiation of hESC. H9-derived hEBs were cultured in the absence (A) or presence (B) of dorsomorphin (DM, 5 µM) and SB431542 (SB, 5∼10 µM) for 4 days, and then attached onto the Matrigel-coated culture dish. After 4 days of adherent culture, the cells were dissociated and analyzed by immunocytochemistry with anti-Pax6 antibody. Quantitative analysis showed that the treatment of DM and SB during EB culture significantly increased the number of Pax6-positive NPs among differentiated cells (C). Scale bar: 50 µm. (TIF) [file pone.0039715.s001.tif]

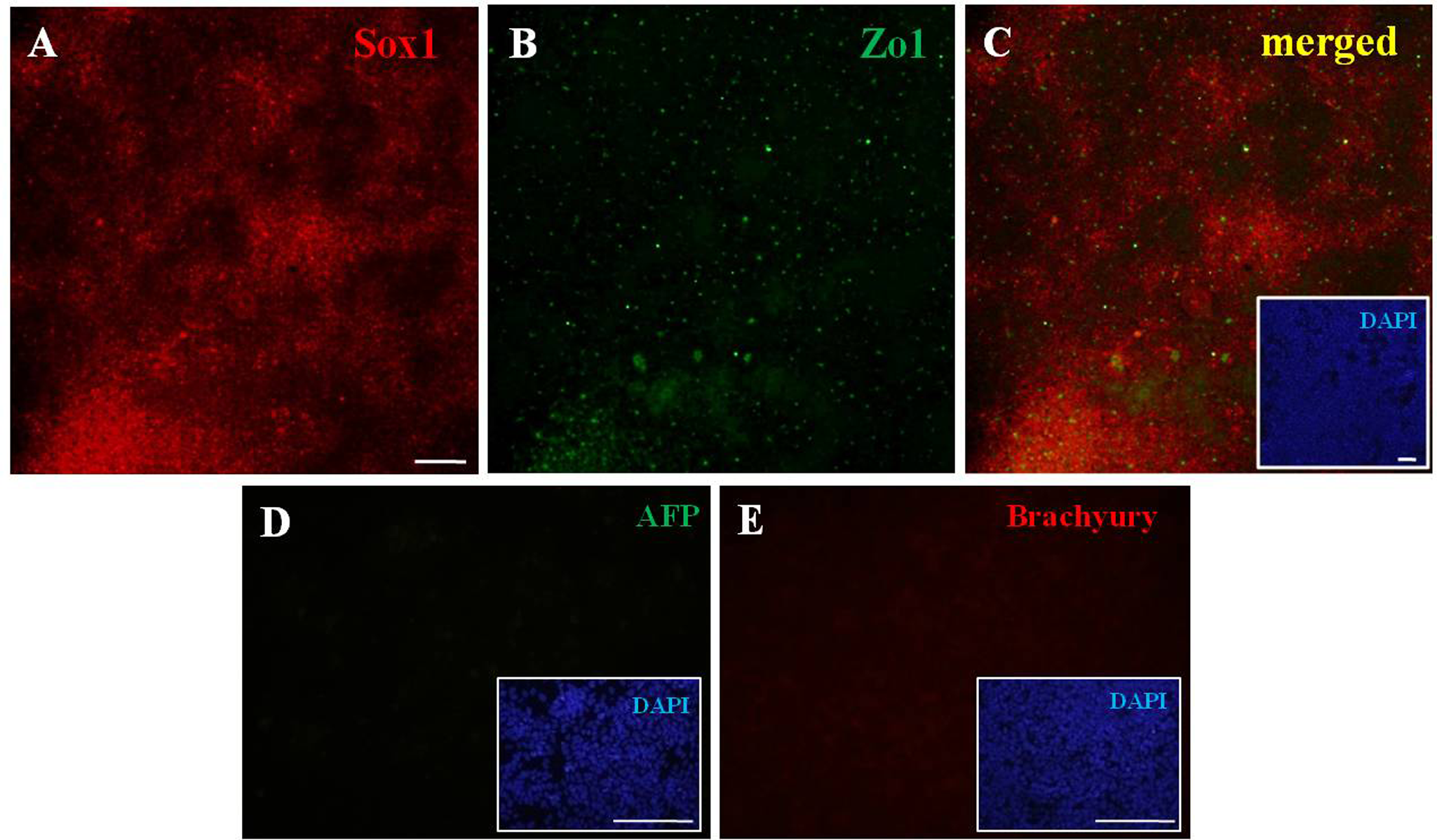

Supplement: Figure S2 — Mechanically isolated neural rosette cells were extensively proliferated in the presence of basic FGF. When hESC-derived neural rosette cells were mechanically isolated and cultured in the presence of basic FGF on culture dishes, the cells vigorously proliferated retaining the expression of neural marker, Sox1 and the typical rosette structure (A–C) devoid of endodermal and mesodermal derivatives (D–E). Scale bars: 100 µm. (TIF) [file pone.0039715.s002.tif]

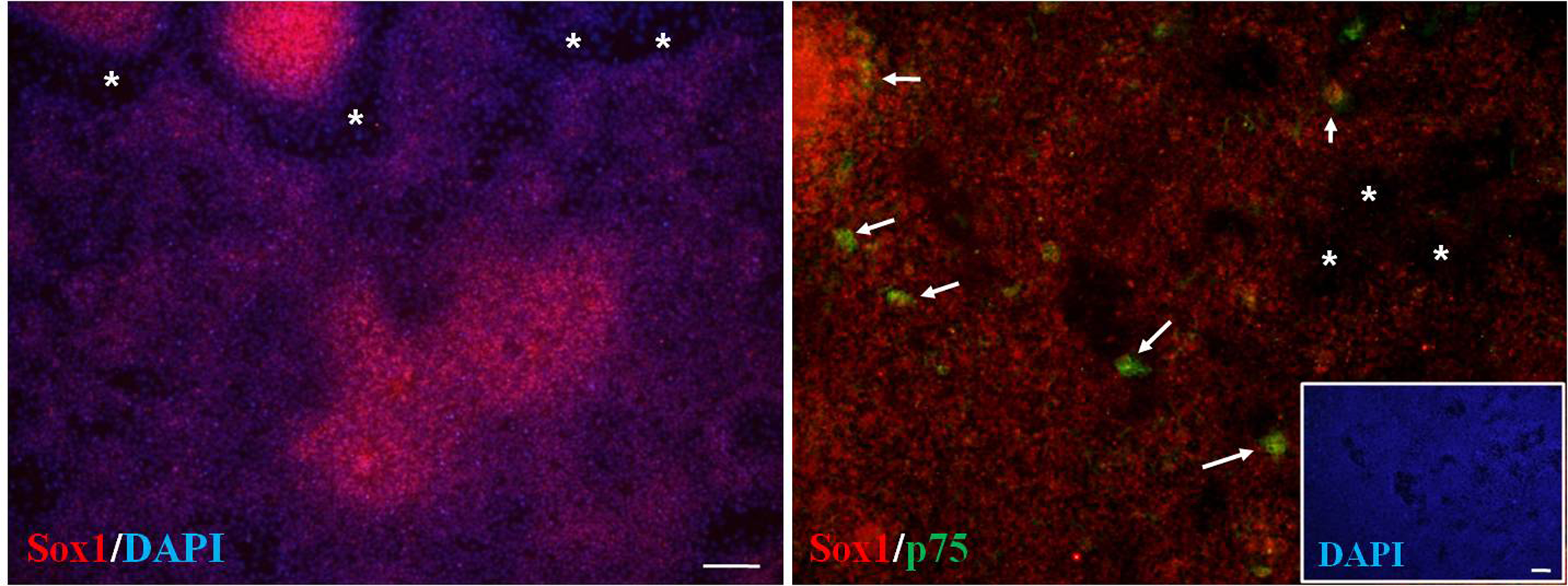

Supplement: Figure S3 — Culture of mechanically isolated neural rosette cells showed the heterogeneity. When mechanically isolated neural rosette cells were expanded on culture dishes, Sox1-negative cells (presumable non-neural cells, indicated by asterisks) and neural crest-lineage precursors (P75-positive cells, indicated by arrows) occasionally appeared in the culture. Scale bars: 100 µm. (TIF) [file pone.0039715.s003.tif]

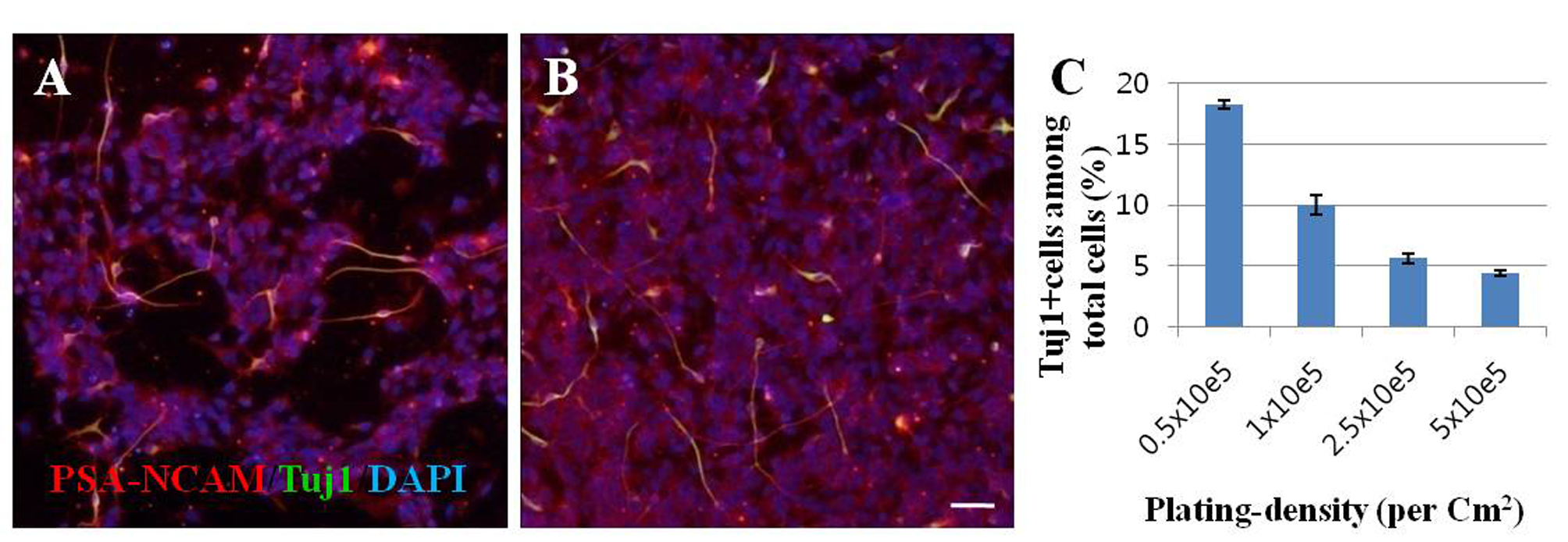

Supplement: Figure S4 — Relationship between the plating density of hNPCPSA-NCAM+ and the occurrence of immature neurons. Relationship between the plating density of hNPCPSA-NCAM+ and the occurrence of immature neurons. (A–B) Representative pictures of immunocytochemical analysis with anti-PSA-NCAM and Tuj1 antibodies at cell density of 1×10^5^ cells/Cm2 (A) and 5×10^5^ cells/Cm2 (B). At low plating-density, Tuj1-positive neuronal progenitors were frequently observed; however, the portion of Tuj1-positive cells significantly diminished as plating density was increasing (C). Scale bar: 50 µm. (TIF) [file pone.0039715.s004.tif]

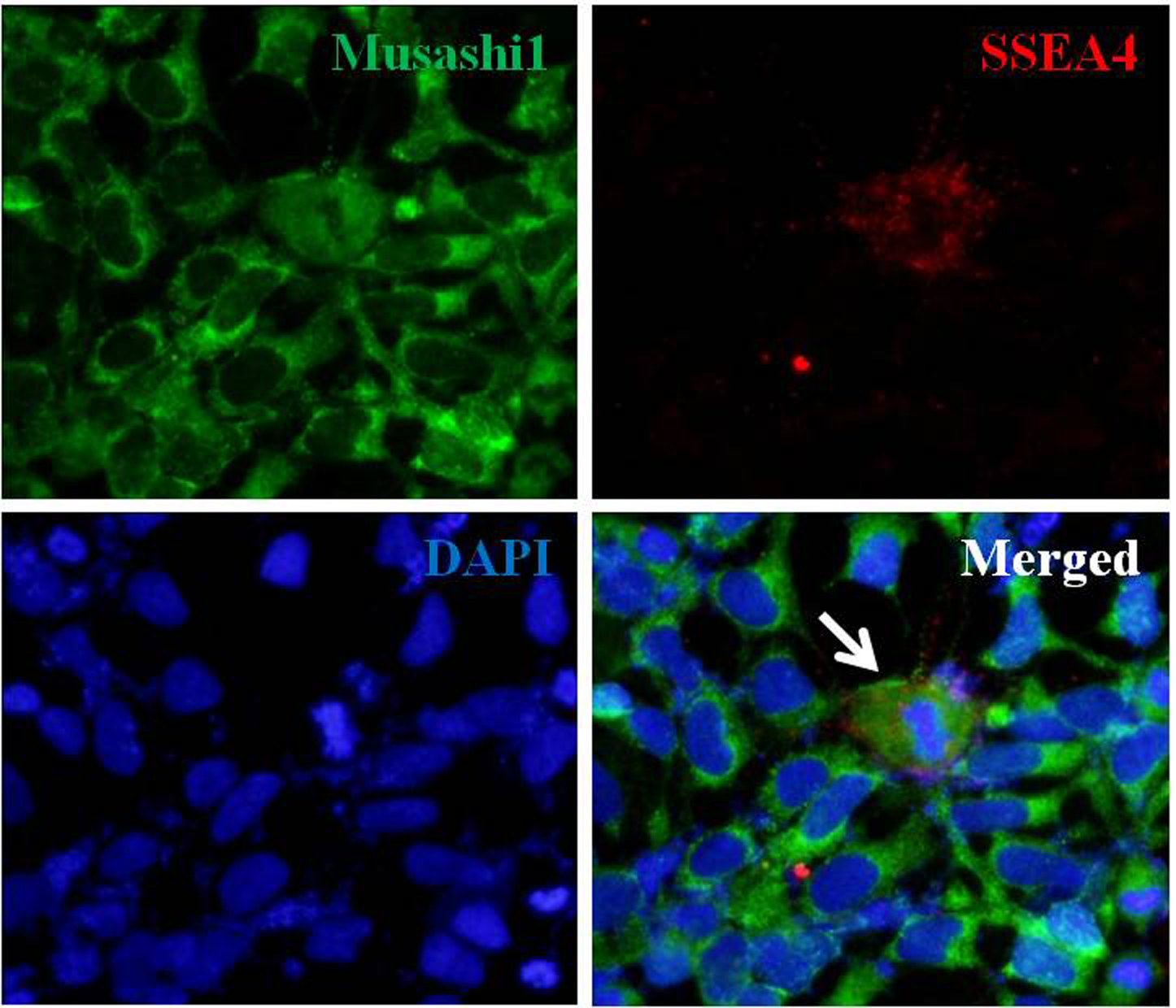

Supplement: Figure S5 — SSEA4-immunoreactive cells in a hNPCPSA-NCAM+ population were in fact the early neuroepithelial cells rather than undifferentiated cells. Immunocytochemical analysis revealed that SSEA4-positive cells in hNPCPSA-NCAM+ culture coexpressed Musashi1 (indicated by arrow) and were under the process of cell division. (TIF) [file pone.0039715.s005.tif]

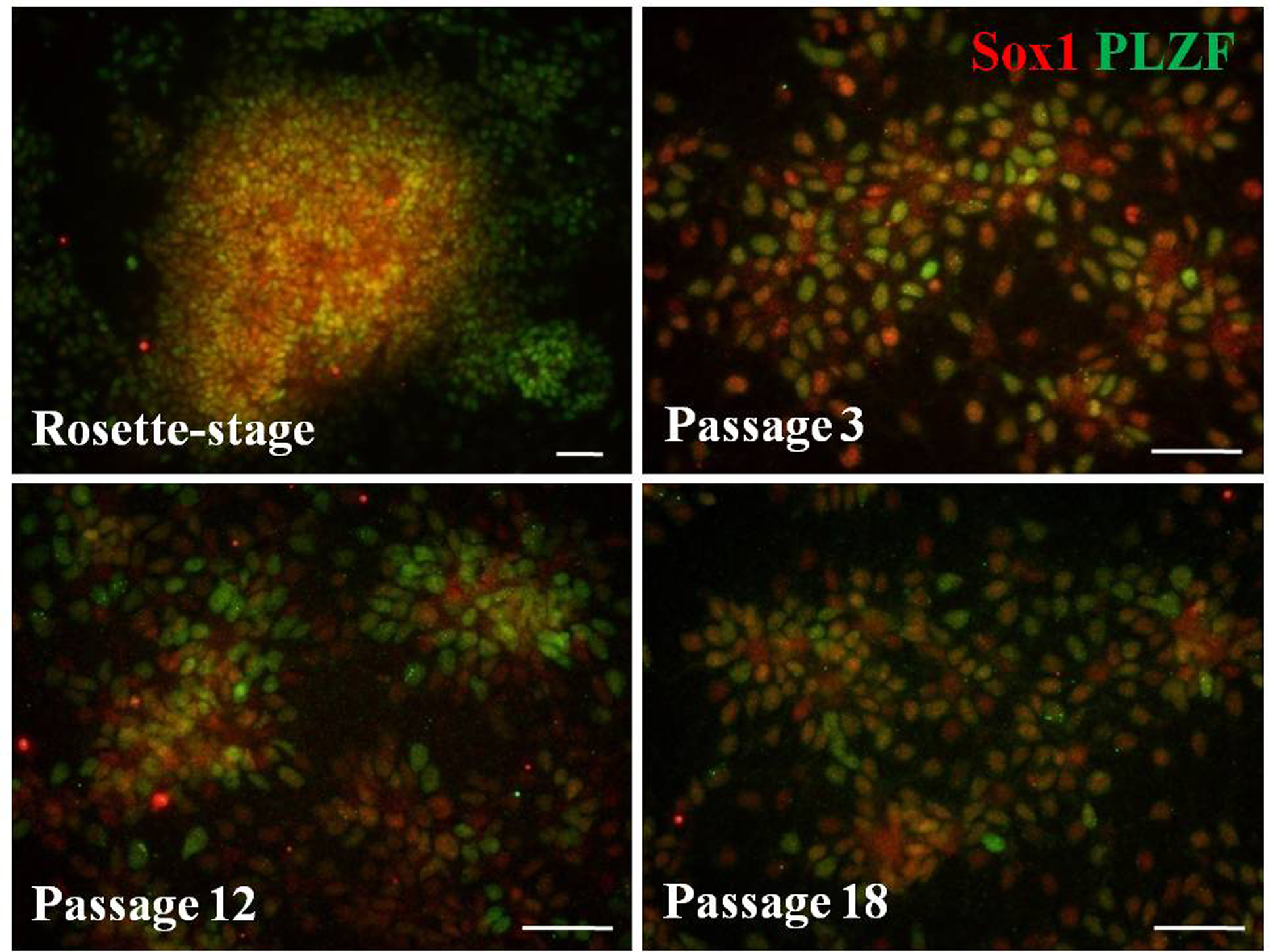

Supplement: Figure S6 — The co-expression of PLZF with Sox1 was maintained from the neural rosette-stage throughout the propagation of hNPCPSA-NCAM+. Scale bars: 50 µm. (TIF) [file pone.0039715.s006.tif]

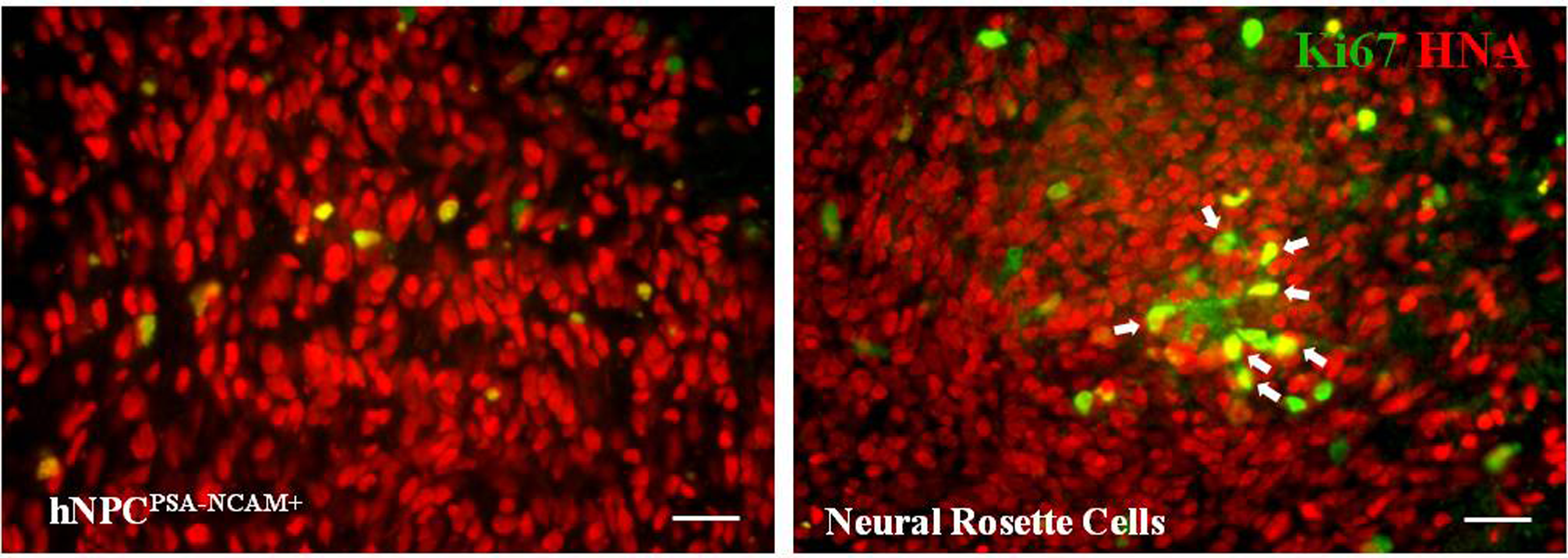

Supplement: Figure S7 — Representative images of hNPCPSA-NCAM+ and neural rosette cell grafts 12 weeks post-transplantation expressing immunoreactivity of HNA and Ki67. While hNPCPSA-NCAM+ graft showed minimal and dispersed Ki67-positive expressions, neural rosette cell graft maintained pronounced expressions of Ki67 and the positive cells showed an apico-basal growth pattern (indicated by arrows). Scale bars: 100 µm. (TIF) [file pone.0039715.s007.tif]
