# Supplementary material for: Highly Pure and Expandable PSA-NCAM-Positive Neural Precursors from Human ESC and iPSC-Derived Neural Rosettes
Source: PLoS One. 2012 Jul 20;7(7):e39715. doi: 10.1371/journal.pone.0039715 (PMC3401209; doi:10.1371/journal.pone.0039715)
Supplement: Tables S1 — Primer sets for semi-quantative PCR (Table S1) and primer sets for real-time PCR (Table S2). (DOCX) [file pone.0039715.s008.docx]

**Table S1. Primer sets for semi-quantative PCR**

| Gene | Forward primer | Reverse primer | Reference |
| --- | --- | --- | --- |
| PST | GCT CCG TTT TGG GGA GAT AGT | CCA CAG AAG ACC CCA GTG ATC | 1 |
| STX | AGC TCT ATT AGA TTT GCT ATG TAA GCT GTT | TCC TCA TCT TCG CAG ACA TCT C | 1 |
| NCAM | CGC TCG GTA CCT GAC CAG AT | GGA ACC CAG TGC ACC TAA GCT | 1 |
| Snail | CTC CTC TAC TTC AGC CTC TT | CTT CAT CAA AGT CCT GTG GG | 2 |
| dHand | AGA AGA CCG ACG TGA AAG AGG AGA | ACA CGG GAG TGT CCT CTT CGT ATT | 2 |
| FoxD3 | CAA GCC CAA GAA CAG CCT AGT GAA | TGA CGA AGC AGT CGT TGA GTG AGA | 2 |
| Sox1 | CAA TGC GGG GAG GAG AAG TC | CTC TGG ACC AAA CTG TGG CG | 3 |
| Pax6 | GGC AAC CTA CGC AAG ATG GC | TGA GGG CTG TGT CTG TTC GG | 3 |
| hTERT | TGG CTG CGT GGT GAA CTT G | GCG GTT GAA GGT GAG ACT GG | 4 |
| PLZF | CTA TGG GCG AGA GGA GAG TG | TCA ATA CAG CGT CAG CCT TG | 4 |
| DACH1 | GTG GAA AAC ACC CCT CAG AA | CTT GTT CCA CAT TGC ACA CC | 4 |
| PLAGL1 | GCC TCA GTC ACC TCA AAA GC | CTT ACC CTG TGG GGC AAA GA | 4 |
| NR2F | ACA GGA ACT GTC CCA TCG AC | GAT GTA GCC GGA CAG GTA GC | 4 |
| PMP2 | CAA GCT AGG CCA GGA ATT TG | CCA CGA CCT TCA TTT TAC AT | 4 |
| HOP | GCA TTG ACA GCT TCA CTC CA | GGA AAT GCT AGC CAC ACC AT | 4 |
| AQP4 | GGA ATT TCT GGC CAT GCT TA | AGA CTT GGC GAT GCT GAT CT | 4 |
| S100β | AAA GAG CAG GAG GTT GTG GA | AGG AAA GGT TTG GCT GCT TT | 4 |
| Actin | GCT CTT TTC CAG CCT TCC TT | CTT CTG CAT CCT GTC AGC AA |  |

**Table S2. Primer sets for real-time PCR**

| Gene | Forward primer | Reverse primer | Reference |
| --- | --- | --- | --- |
| TH | CTG TGG CCT TTG AGG AGA AG | ATG GTG GAT TTT GGC TTC AA | - |
| En1 | GCCCGT GGT CAA AAC TGA | TTC TTC TTC AGC TTC CTG GTG | - |
| Nurr1 | CGA AAA CGC CTG TAA CTC G | TCT CCC GAA GAG TGG TAA CTG | - |
| Pitx3 | AGA TCT GCC TGT TGC AGG A | CAG CGT CTG ACA GCG ACA | - |
| Lmx1a | GAG ACC ACA TGC ACC CTT ATG | GGT TAC TGA GGG AGG TGT CG | - |
| Lmx1b | GGA AGA GGG AGA TAA CTG TGT GGT | CCT GGG GCA ATT ATT TCC TCT TTC TG | - |
| FoxA2 | TGT CTG AGG AGT CGG AGA GC | ACC GCT CCC AGC ATA CTT T | - |
| Actin | GCT CTT TTC CAG CCT TCC TT | CTT CTG CAT CCT GTC AGC AA | - |

Supplementary Table References

1. Mayanil CS, George D, Mania-Farnell B, Bremer CL, McLone DG, and Bremer EG. Overexpression of murine Pax3 increases NCAM polysialylation in a human medulloblastoma cell line. *J Biol Chem.* 2000;275:23259-66.

2. Pomp O, Brokhman I, Ben-Dor I, Reubinoff B, Goldstein RS.Generation of Peripheral Sensory and Sympathetic Neurons and Neural Crest Cells from Human Embryonic Stem Cells *Stem Cells* 2005;23;923-930

3. Cho MS, Lee YE, Kim JY, et al. Highly efficient and large-scale generation of functional dopamine neurons from human embryonic stem cells. *Proc Natl Acad Sci U S A*. 2008;105:3392-7

4. Koch P, Opitz T, Steinbeck JA, Ladewig J, Brüstle O. A rosette-type, self-renewing human ES cell-derived neural stem cell with potential for in vitro instruction and synaptic integration. *Proc Natl Acad Sci U S A.* 2009;106:3225-30.
